# Syndecan-4 as a biomarker to predict clinical outcome for glioblastoma multiforme treated with WT1 peptide vaccine

**DOI:** 10.4155/fsoa-2015-0008

**Published:** 2016-10-03

**Authors:** Satoshi Takashima, Yoshihiro Oka, Fumihiro Fujiki, Soyoko Morimoto, Hiroko Nakajima, Yoshiki Nakae, Jun Nakata, Sumiyuki Nishida, Naoki Hosen, Naoya Tatsumi, Kenji Mizuguchi, Naoya Hashimoto, Yusuke Oji, Akihiro Tsuboi, Atsushi Kumanogoh, Haruo Sugiyama

**Affiliations:** 1 Department of Respiratory Medicine, Allergy & Rheumatic Diseases, Osaka University Graduate School of Medicine, 2-2 Yamadaoka, Suita City, Osaka 565-0871, Japan; 2 Department of Cancer Immunology, Osaka University Graduate School of Medicine, 1-7 Yamadaoka, Suita City, Osaka 565-0871, Japan; 3 Department of Immunopathology, Immunology Frontier Research Center (World Premier International Research Center), Osaka University, 3-1 Yamadaoka, Suita City, Osaka 565-0871, Japan; 4 Department of Hematology, Kitano Hospital, 2-4-10 Ohgimachi, Kita-ku, Osaka City, Osaka 530-8480, Japan; 5 Department of Cancer Immunotherapy, Osaka University Graduate School of Medicine, 2-2 Yamadaoka, Suita City, Osaka 565-0871, Japan; 6 Department of Cancer Stem Cell Biology, Osaka University Graduate School of Medicine, 1-7 Yamadaoka, Suita City, Osaka 565-0871, Japan; 7 National Institutes of Biomedical Innovation, Health & Nutrition 7-6-8 Saito-Asagi, Ibaraki City, Osaka 567-0085, Japan; 8 Department of Neurosurgery, Kyoto Prefectural University Graduate Schoolof Medicine, 465 Kajii-cho, Hirokoji-agaru, Kawaramachi-dori, Kamigyo-ku, Kyoto City, Kyoto 602-8566, Japan; 9 Department of Neurosurgery, Graduate School of Medicine, Osaka University, 2-2 Yamadaoka, Suita City, Osaka 565-0871, Japan

**Keywords:** cancer immunotherapy, DC-HIL, GBM, glioblastoma multiforme, immune checkpoint inhibitor, malignant glioma, peptide vaccine, SDC-4, Syndecan-4, WT1

## Abstract

**Aim:**

In cancer immunotherapy, biomarkers are important for identification of responsive patients. This study was aimed to find biomarkers that predict clinical outcome of WT1 peptide vaccination.

**Materials & methods:**

Candidate genes that were expressed differentially between long- and short-term survivors were identified by cDNA microarray analysis of peripheral blood mononuclear cells that were extracted from 30 glioblastoma patients (discovery set) prior to vaccination and validated by quantitative RT-PCR using discovery set and different 23 patients (validation set).

**Results:**

*SDC-4* mRNA expression levels distinguished between the long- and short-term survivors: 1-year survival rates were 64.0 and 18.5% in *SDC4*-low and -high patients, respectively.

**Conclusion:**

*SDC-4* is a novel predictive biomarker for the efficacy of WT1 peptide vaccine.

## Lay abstract

Recently, cancer immunotherapies are becoming a standard therapeutic option. To improve their efficacy, identification of biomarkers is important to select responsive patients. In this study, we identified *SDC-4* as a biomarker to predict clinical outcome using peripheral blood mononuclear cells obtained from patients with glioblastoma, a malignant brain tumor, who were treated with WT1 peptide vaccine. With 30 samples of peripheral blood mononuclear cells prior to vaccination, 32 candidate genes were filtrated by microarray, and finally only *SDC-4* was validated by RT-PCR using another 23 samples. Accordingly, 1-year survival rates were 64.0 and 18.5% in *SDC4*-low and -high patients, respectively.

**Figure 1.**
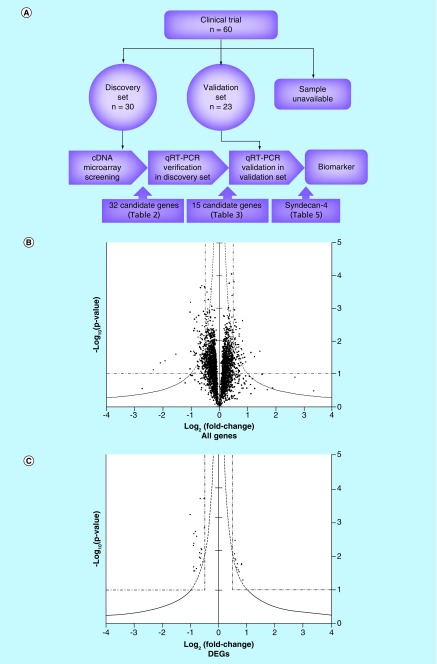
**Selection of candidate genes by cDNA microarray analysis.** **(A)** Strategy to find biomarkers is schematically shown. First, DEGs were screened by cDNA microarray and the expression levels of screened genes were verified by quantitative RT-PCR using 30 glioblastoma multiforme patients in a discovery set. Second, these verified DEGs were validated using different 23 GBM patients in a validation set. Finally, only *SDC-4* was identified as a biomarker. **(B)** A volcano plot was generated. Each dot corresponds to one gene. X- and y-axes indicate fold change (log_2_[short/long]) of signal intensities of individual genes and the statistical significance (-log_10_[p-value]) of the difference in the signal intensities of individual genes between long- and short-term survivors, respectively. Dashed line indicates y = |x|^–1^, y = 1 and |x| = 0.5. **(C)** Thirty-two candidate DEGs were extracted as described in the text. Gene names and their statistical evaluations are shown in Table 2. DEG: Differentially expressed gene.

**Figure 2.**
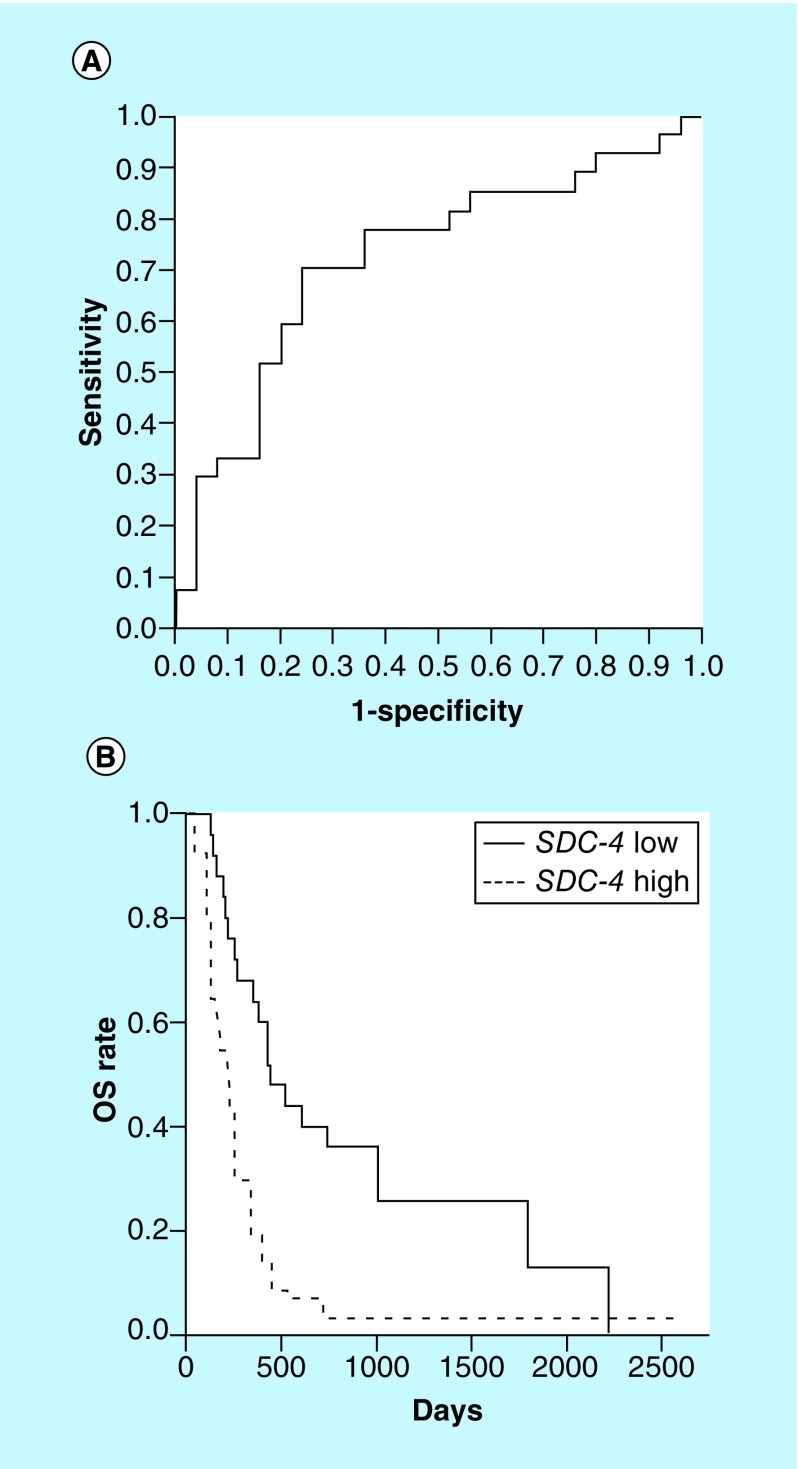
***SDC-4* is a prediction marker for overall survival.** **(A)** AUC-maximized receiver operating characteristic curve was generated. Optimal cut-off value, 0.001 of *SDC-4* relative expression levels (log_2_[1 + 2^−ΔCT^]) discriminates between long-term (OS ≥256 days) and short-term (OS <256 days) survivors, and *SDC-4* expression levels in long-term survivors are ≤0.001. Statistical capabilities are 70.4% sensitivity, 76.0% specificity, 76.0% positive predictive value, 70.4% negative predictive value and 73.1% accuracy, and a Chi-square test shows a statistical significance (p < 0.001). All the 53 patients in the discovery and validation sets are included in this analysis. **(B)** Kaplan–Meier curves of OS (days) of patients with ≤0.001 (*SDC-4* low) and >0.001 (*SDC-4* high) of *SDC-4*-expression levels are shown. Comparison of OS between the two groups is performed using a two-sided log-rank test, and the difference in OS is statistically significant (p < 0.001). One-year OS rates were 64.0 and 18.5% in *SDC-4*-low and -high patients, respectively. OS: Overall survival.

WT1, a transcription factor, regulates many kinds of important genes that play important roles in embryogenesis, cell proliferation and differentiation [1–3]. The *WT1* gene is overexpressed in leukemia and a variety of solid tumors, in which it exerts an oncogenic function [4,5]. WT1, a pan-tumor-associated antigen (TAA), was identified as the best one among 75 TAAs, based on criteria including therapeutic function, immunogenicity, oncogenicity, specificity, expression levels and percent of positive cells, expression levels in stem cell, number of patients with antigen-positive cancers, number of epitopes and cellular localization [6]. Our group and others have performed WT1-targeted cancer immunotherapy, including WT1 peptide vaccination, and WT1 peptide-pulsed [7] or *WT1* mRNA-electroporated [7–9] dendritic cell (DC) therapy, and obtained a series of successful results with positive immunological and clinical responses in patients with glioblastoma multiforme (GBM) [10–14], acute myeloid leukemia [15–19], chronic myeloid leukemia [20,21], myelodysplastic syndromes [22–24], multiple myeloma [25], malignant melanoma [26], infantile rhabdomyosarcoma [27], and lung [28], breast [15], pancreatic [29,30], ovarian [31,32], uterine [23,33] and salivary gland cancers [34,35].

GBM is a malignant brain tumor with very poor prognosis. The standard therapy for the newly diagnosed GBM is surgery, followed by irradiation and chemotherapy. However, a 5-year survival rate is less than 10%, and once recurrence occurs, therapeutic options are limited [36]. In 2008, we reported promising results from a Phase II clinical study of WT1 peptide vaccination in patients with recurrent or conventional therapy-resistant GBM. In patients who received the vaccination, progression-free survival (PFS) at 6 months was 33.3%, and the median overall survival (OS) was 36.7 weeks, suggesting the therapeutic potential of WT1 peptide vaccine for GBM patients [10].

In order to improve the clinical usefulness of WT1 peptide vaccine, it is crucial not only to biologically enhance the vaccine's efficacy but also to select patients who are likely to respond to the vaccine. Identification of responders would be facilitated by the availability of reliable biomarkers that predicted the clinical outcome of patients treated with the WT1 peptide vaccine.

To date, several studies by our group and others have identified markers that are correlated with the clinical efficacy of WT1-based immunotherapy. Malignant glioma patients with higher WT1 expression levels (score 3–4) lived longer after immunotherapy than those with lower expression levels (score 1–2) [13]. An increase in the frequency of WT1-specific cytotoxic T lymphocytes (CTLs) before and after WT1 peptide vaccination was correlated with clinical response [15,17,30]. Upon repeated vaccination with WT1 peptide, an increase in the frequencies of effector memory subsets, which are important for maintenance of WT1-specific CTLs, was also identified as a predictor of responders [30]. Lymphocyte numbers in peripheral blood and antigen-specific delayed-type hypersensitivity (DTH) also predict clinical outcome in various immunotherapies [37]. However, no reliable biomarkers have yet been established that can predict the clinical outcome of immunotherapies, such as TAA-targeting cancer vaccines prior to therapy. High-throughput technologies, such as cDNA microarray analysis, have been investigated as approaches to discover biomarkers for prediction of clinical outcomes of therapeutic interventions.

In this study, we used cDNA microarrays to comprehensively analyze gene-expression profiles of peripheral blood mononuclear cells (PBMCs) with the goal of identifying biomarkers that predicted the outcome of WT1 peptide vaccination in patients with recurrent or conventional therapy-resistant GBM. The results revealed that *SDC-4* mRNA expression levels prior to WT1 vaccination were a promising predictive biomarker for clinical outcome in these patients.

## Materials & methods

### WT1 peptide vaccine

The WT1 peptide vaccine consists of WT1-CTL epitope peptide and Montanide ISA51 adjuvant. The peptide used in this study is a modified 9-mer WT1 peptide (amino acids [aa] 235–243 CYTWNQMNL; mWT1–235), in which M was replaced by Y at the second amino acid position, an anchor position for HLA-A*24:02, of the natural WT1–235 peptide (235–243 CMTWNQMNL; nWT1–235). The binding affinity of mWT1–235 for HLA-A*24:02 is higher than that of nWT1–235, and mWT1–235 induced a much stronger CTL response against WT1-expressing tumor cells. GMP-grade WT1 peptide was purchased from Multiple Peptide Systems (CA, USA) and Peptide Institute (Osaka, Japan) as the lyophilized peptide.

### Patients

Sixty patients were enrolled in a Phase II study of WT1 peptide vaccination of GBM patients, the result of which we reported previously [10,13]. Patients with recurrent or progressive GBM were eligible to be enrolled in the Phase II study if their disease was resistant to conventional chemotherapy or radiotherapy. Other inclusion criteria were as described in our previous study. Briefly, they were: first, age between 16 and 80 years; second, expression of WT1 in glioma cells as determined by immunohistochemical analysis; third, HLA-A*2402–positivity; fourth, estimated survival of more than 3 months; fifth, ECOG Performance Status Grade 0–2; sixth, no severe organ function impairment and seventh, written informed consent of the patient. All enrolled patients were histologically proven to have GBM (Grade 4) based on the WHO criteria.

PBMC samples obtained prior to WT1 vaccination were available from 53 of the 60 patients enrolled in the Phase II study; all 53 were used in this study. Thirty of the patients were randomly assigned to the discovery set, and the remaining 23 were assigned to the validation set.

The median OS of the patients in the discovery set was 347 days from the start of the vaccination and 460 days from the time of recurrence or disease progression to death or censored time points. The median PFS, which was defined as the days from the start of the vaccination to the disease progression, was 62 days. In the microarray analysis, to select differentially expressed genes (DEGs) that influence not only short-term outcome (RECIST, PFS and OS from the time of vaccination started) but also long-term outcome, the 30 patients in a discovery set were divided into two groups with OS from the time of recurrence or disease progression. The 15 patients with OS of ≥460 days were defined as long-term survivors (OS median: 1133 days, range: 480–2678 days), whereas the remaining 15 patients with OS of <460 days were defined as short-term survivors (OS median: 216 days, range: 138–458 days) (Table 1).

In the verification process using discovery set, OS was the days from the first vaccination to death or censored time points which should more strictly reflect the vaccination effect. Thus, in the verification step, patients who survived ≥347 days (the median OS of the discovery set according to the immediately preceding definition) were defined as long-term survivors, whereas those who survived <340 days were defined as short-term survivors.

In the validation set, median OS from the first vaccination was 257 days. The 12 patients who survived ≥256 days were defined as long-term survivors, and the 11 patients who survived <256 days were defined as short-term survivors.

All short-term survivors in the discovery and validation sets died before a censored time point. Therefore, patient categorization of long- and short-term survivors was fixed (Supplementary Table 1). The Phase II study of WT1 peptide vaccination and cDNA microarray analysis of the blood samples was approved by the ethical review board of Osaka University Hospital.

### WT1 peptide vaccination schedule

After informed consent was obtained, weekly intradermal injection of 3.0 mg of HLA-A*24:02–restricted mWT1–235 peptide emulsified with Montanide ISA51 adjuvant was initiated. Vaccinations were scheduled for 12 consecutive weeks, after which responses were evaluated by MRI. Responses were classified as complete response, partial response, stable disease and progressive disease using MRI according to the RECIST (Response Evaluation Criteria In Solid Tumors) criteria. When clinical response was obvious, WT1 vaccination was continued at 2-week intervals for the next few months, and then at 1–3-month intervals until obvious tumor progression or deterioration of the patient's condition was observed.

### Blood samples

Peripheral blood was obtained from patients immediately before the first vaccination, followed by separation of PBMCs by density gradient centrifugation using Lymphocyte Separation Solution (Nacalai Tesque, Kyoto, Japan). Separated PBMCs were stored in liquid nitrogen prior to use.

### RNA isolation from PBMCs & cDNA microarray analysis

Frozen PBMCs were thawed, and total RNA was isolated using the TRIzol reagent (Life Technologies, CA, USA), purified using chloroform and subjected to isopropanol and ethanol precipitation. Purified total RNA was quantitated using a NanoDrop ND-1000 (Thermo Fisher Scientific, MA, USA).

RNAs from PBMCs of the discovery set patients were sent to Toray Industries (Tokyo, Japan), which performed RNA-based cDNA microarray analysis using Human oligo chip 25k ver1.00 (Toray Industries).

Microarray analysis data were transformed using global normalization, followed by quantile normalization [38]. First, genes with intensities less than ([average intensities of blank spots] + 10 × [standard deviation of intensities of blank spots]) were excluded. To identify DEGs between long- and short-term survivors, a volcano plot of -log_10_(p-value) of Welch's t-test between the two groups (y-axis) versus log_2_(fold change) (x-axis) was made [39]. The fold change between the two groups was calculated as log_2_([mean signal intensities in long-term survivors]/[mean signal intensities in short-term survivors]). DEGs were selected using this volcano plot according to the following conditions: y > |x|^−1^, |x| >0.5 and y >1.0. To enrich for DEGs, correlations between individual gene expression intensities and either PFS or clinical responses according to RECIST criteria were examined by Pearson's and Spearman's correlation coefficient, respectively. Genes that did not exhibit significant p-values (alpha level 0.1 for Pearson's correlation coefficient and 0.2 for Spearman's correlation coefficient) were excluded from further analysis. In this series of microarray analyses, a nonstringent cut-off for the p-value was set according to MAQC guidelines [40]. Finally, genes that satisfied the following six conditions were retained for further analysis: first, average intensity ≥([average intensities of blank spots] + 10 × [standard deviation of intensities of blank spots]); second, -log_10_(p-value of difference between long- and short-OS groups) was greater than |log_2_(fold difference)|^–1^; third, |log_2_(fold difference)| was greater than 0.5; fourth, -log_10_ (p-value of difference between long- and short-OS groups) greater than 1.0; fifth, p-value of Pearson's correlation between PFS and signal intensities less than 0.1; and sixth, p-value of Spearman's correlation between clinical outcome and signal intensities less than 0.2.

### RT-PCR

RNAs from PBMCs were reverse-transcribed into first-strand cDNA using SuperScript VILO cDNA Synthesis Kit and Master Mix (Life Technologies). Sequences of primers used for RT-PCR are provided in Supplementary Table 2. Target sequences are available from the NCBI nucleotide database [41]. Sample cDNAs were preamplified with dilute primer mixture for 15 cycles prior to loading to the BioMark 48.48 dynamic array nanofluidic chips (Fluidigm, CA, USA). RT-PCR was performed on a BioMark HD (Fluidigm). Regarding internal control genes, we quantified many control genes, including *ACTB*, *GAPDH*, *RPL13*, *RPL18A*, *PPIA*, *TSR2* and *RNA28S1*. Each control gene was validated in comparison with each other, and correlation matrix was generated, followed by scoring each gene. Consequently, *ACTB* was determined as the most stable internal control gene among all the candidate control genes and was used as the internal control. Then, ΔCTs were calculated as ([CT value of each genes] – [CT value of *ACTB*]). All data concerning gene expression levels were converted to log_2_(1 + 2^−ΔCT^), and the relative expression levels were statistically analyzed.

### Statistical analysis

Cox proportional hazard regression models were used to evaluate associations between patient characteristics and OS in the discovery and validation sets. In the microarray analysis, PFS was logarithmically transformed to yield a normal distribution.

In statistical analysis of the result from RT-PCR, the Jarque–Bera test was used to assess normality of variable distributions, and the F-test was used to test homogeneity of variance.

To promote statistical power, parametric analyses were conducted as much as possible. For examination of the difference between long- and short-term survivors, an appropriate transformation (e.g., logarithmic, square-root, cube-root or fourth-root transformation) was performed for individual genes to satisfy the requirement for normality. All normally distributed variables were checked for homogeneity of variance and analyzed by Student's t-test. Non-normally distributed variables were analyzed using the Mann–Whitney U-test.

The *SDC-4* expression levels that discriminated responders and nonresponders (*SDC-4* cut-off value) were determined using a receiver operating characteristic (ROC) curve in order to maximize the Youden's index (defined as Youden's J statistic = sensitivity + specificity -1). The ROC curve was drawn based on the cut-off value of OS, which maximized area under the ROC curve (AUC). The horizontal and vertical axes indicate 1-specificity and sensitivity for responder, respectively. Sensitivity, specificity, positive and negative predictive values, and accuracy of the prediction of OS by the *SDC-4* expression levels were calculated using standard formulas. Kaplan–Meier curves and a two-sided log-rank test were used to assess differences between two groups defined by the *SDC-4* cut-off value. Statistical analysis was conducted using appropriate software including JMP Pro 10.0 (SAS Institute, Inc., NC, USA) and R-commander [42].

## Results

### Identification of DEGs in PBMCs prior to WT1 vaccination

Patients with recurrent or conventional therapy-resistant GBM who were treated with WT1 peptide vaccine were selected as the cohort for this analysis. PBMCs were obtained prior to WT1 vaccination from 53 of 60 patients in this vaccination trial. The strategy to search useful biomarkers for prediction of clinical outcome is shown schematically in Figure 1A. Patients were assigned randomly into the discovery and validation sets (30 and 23 patients, respectively); within the validation set, patients were classified as long-term or short-term survivors (15 patients each) (Table 1). Cox proportional hazard regression models revealed that within the discovery set, there was no association between OS following vaccination and age, gender, presence or absence of prior surgical treatment, or prior total absorbed dose of radiotherapy; however, low-performance status and history of chemotherapy were indicators of worse clinical outcome (Table 1). We discussed these issues that affect clinical outcome later in the last part of the Results and Discussion section.

To identify candidate genes that were expressed differentially between long- and short-term survivors, we performed cDNA microarray analysis on PBMCs. A volcano plot of statistical significance (-log_10_ p-value) versus log_2_ (fold change), in which each dot indicates one of a total of 25,000 genes, is shown in Figure 1B. DEGs were extracted as follows. First, we excluded genes with intensities less than ([average intensities of blank spots] + 10 × [standard deviation of intensities of blank spots]). The remaining 3037 genes were filtrated to a set of 74 genes that satisfied the following three criteria, y > |x|^–1^, |x| > 0.5 and y > 1.0. To further enrich for candidate genes, the correlation between the signal intensities of individual genes and either PFS or clinical response was evaluated by Pearson's correlation and Spearman's rank correlation analysis, respectively, and genes with p-values ≥ 0.1 (Pearson's) or ≥ 0.2 (Spearman's) were excluded. Ultimately, 32 genes were selected as candidates (Table 2 & Figure 1C). Of these candidate genes, 11 were highly expressed in long-term survivors (Table 2, upper); and 21 were highly expressed in short-term survivors (Table 2, lower).

### Verification of candidate genes by quantitative RT-PCR

Expression levels of the 32 candidate genes identified in the cDNA microarray analysis of the patients in the discovery set were verified by quantitative RT-PCR.

In order to ensure a rigorous statistical analysis, individual gene expression levels obtained by quantitative RT-PCR were transformed appropriately to satisfy the requirement for normality. Distributions were checked for normality by the Jarque–Bera test, for homogeneity of variance by the F-test, and for significance by one-tailed Student's t-test (Table 3). When no transformation yielded a sufficiently normal distribution, the one-tailed Mann–Whitney U-test was used. Because one-tailed tests were used, genes with fold changes that were inverted relative to those obtained from the microarrays were automatically excluded. As a result of these analyses, 15 genes whose expression levels correlated to OS were retained, and 17 were excluded (Table 3).

### Verification of 15 candidate genes using patients in the validation set

Next, we prepared a validation set consisting of 23 GBM patients treated with WT1 peptide vaccine following the same protocol used in the discovery set. Characteristics of patients in the validation set are provided in Table 4. No association was detected between OS and age, gender, performance status, the presence or absence of prior surgical treatment and chemotherapy, or prior total absorbed doses on radiotherapy. Using data from the patients in the validation set, we investigated whether the expression levels of the 15 candidate genes identified in the discovery set correlated with OS using the same statistical methods as those used in the discovery set. Only expression of *SDC-4* significantly (negatively) correlated with OS in the validation set (Table 5).

### Prognostic prediction of GBM patients treated with WT1 peptide vaccine using *SDC-4* expression levels

First, based on the values of OS and *SDC-4* expression levels, we generated a ROC curve using all 53 patients from the discovery and validation sets (Figure 2A). Generally, area under the ROC curve (AUC) of an efficient biomarker shows high AUC, maximum of which is 1.0. Since AUC varies in accordance with cut-off values of OS, the cut-off value was selected in order to maximize the AUC; therefore, the patients were classified into two groups, ‘responders’ and ‘nonresponders’ by cut-off value of 256 days and then AUC was 0.72. The best cut-off value of *SDC-4* expression levels to classify responders (OS of ≥256 days) and nonresponders (OS of <256 days) was determined to be 0.001 according to the AUC-maximized ROC curve using the Youden's index; *SDC-4* expression levels ≤0.001 and >0.001 predicted responders and nonresponders, respectively. Prediction of likely responders had 70.4% sensitivity, 76.0% specificity, 76.0% positive prediction value, 70.4% negative prediction value and 73.1% accuracy; in addition, a Chi-square test demonstrated a significant difference (p < 0.001).

Furthermore, we performed survival analysis using this cut-off value of *SDC-4* expression level (0.001). The patients were divided into two groups, *SDC-4*-low (*SDC-4* ≤ 0.001) and -high (*SDC-4* > 0.001) groups (Figure 2B). The difference in OS between the two groups was estimated by Kaplan–Meier curves and the subsequent log-rank test. The results revealed that *SDC-4*-low patients survived significantly longer than *SDC-4*-high patients; 1-year OS rates were 64.0 and 18.5%, respectively (Figure 2B).

As shown in Table 1, low-performance status and the past history of chemotherapy were indicated to be risk factors of worse clinical outcome for the discovery set patients. Therefore, potential confounding effects of *SDC-4* expression levels and baseline patient characteristics, including the two factors mentioned above, were assessed using Cox proportional hazard regression models in all the 53 patients (Supplementary Table 3). This analysis allowed us to assess the association between *SDC-4* expression levels and the risk of death at any given time points while controlling for other predictors that may affect the risk of death. As a result, it was suggested that high SDC-4 expression level of *SDC-4* was a significant risk factor for the worse clinical outcome (HR: 13.8; 95% CI: 1.35–84.2; p = 0.027) and a predictor of the clinical outcome independent of other risk factors.

## Discussion

In this study, we demonstrated that *SDC-4* expression levels in PBMCs obtained prior to WT1 vaccination were significantly and negatively correlated with OS of recurrent or conventional therapy-resistant GBM patients who were treated with WT1 peptide vaccine, and that *SDC-4* expression levels were a useful biomarker for prediction of clinical outcome.

T cells are a major player in tumor immunity. T cells are activated through an interaction between T-cell receptors and antigen/MHC molecule complexes on antigen-presenting cells (APCs); that is regulated in a costimulatory or coinhibitory manner by accessory receptors. A costimulatory signal is transmitted by the interaction between CD28 receptor on T cells and CD80 or CD86 on APCs. On the other hand, coinhibitory signals are transmitted by a variety of molecules, including the interactions among CTLA-4 (cytotoxic T lymphocyte antigen-4) on T cells for CD80 and CD86 on APCs; programmed cell death-1 (PD-1) and its ligands PD-L1 and PD-L2; B- and T-lymphocyte attenuator and herpes virus entry mediator; Tim-3 (T cell immunoglobulin- and mucin domain-containing molecules 3) and Tim-3L [43,44]; and by TIGIT (T cell immunoreceptor with immunoglobulin and ITIM domain) [45]. SDC-4 is a novel type of coinhibitor distinct from those listed above.

Syndecans are type-I transmembrane heparan sulfate proteoglycans that bind to the extracellular matrix and a variety of cytokines, chemokines and growth factors, thereby modifiying their local concentration, stability and accessibility to their respective receptors; consequently, they significantly influence cell proliferation and differentiation. Mammals have four known syndecans, (SDC-1, -2, -3 and -4), of which only SDC-4 is expressed ubiquitously; expression of the other three is tissue-restricted [46]. SDC-4 is upregulated on activated T cells via activation of NF-κB [47]. In addition, it interacts with DC-associated heparan sulfate proteoglycan-dependent integrin ligand (DC-HIL) and thus mediates the coinhibitory effect of DC-HIL on T-cell activation [43]. Knockdown of SDC-4 expression enhances the T-cell response to APCs, and blockage of endogenous SDC-4 using specific antibodies or soluble SDC-4 receptor enhances T-cell reactions to syngeneic and allogeneic stimulation *in vitro* and exacerbated contact hypersensitivity responses *in vivo* [48]. Consistent with this, transplantation of SDC-4^-/-^ T cells into sublethally γ-irradiated allogeneic mice induces hyperproliferation of infused T cells [48]. Collectively, these observations indicate that SDC-4 is the T-cell ligand through which DC-HIL mediates its coinhibitory function. On the other hand, DC-HIL also binds to SDC-4 on activated T cells and is expressed most strongly by epidermal Langerhans cells, an immature type of DC [49]. DC-HIL expression levels on CD14^+^ monocytes inversely correlate with allostimulatory capacity, and knockdown of DC-HIL enhances allostimulation. Deletion of DC-HIL abrogates the T-cell suppressor activity of myeloid-derived suppressor cells (MDSCs) [50]. These observations clearly suggest that the SDC-4/DC-HIL pathway is one of the most important factors in regulation of immune responses mediated by T cells, APCs and MDSCs. Most importantly, downregulation of either or both SDC-4 and DC-HIL augments T-cell-mediated-immune responses by attenuating the interaction between the two molecules and/or reducing the T-cell suppressor function of MDSCs [50].

Based on the findings described above, our present results might be interpreted as follows. Low expression of *SDC-4* in PBMCs, which correlated with favorable clinical effects of WT1 peptide vaccination of GBM patients, reflected downregulated expression of SDC-4 on T cells. In addition, this downregulation of SDC-4 augmented WT1-specific immune responses to WT1 peptide vaccination by attenuating the interaction between SDC-4 and DC-HIL on monocytes (DCs), leading to improved clinical effects.

On the other hand, it was also reported that, besides T-cell activation state, various pathophysiological conditions, including bacterial endotoxin shock [51], acute pneumonia [52], *Helicobacter pylori* infection [53], atherosclerosis [54] and ischemic heart disease [55], are associated with SDC-4 expression levels in PBMCs. Therefore, we need to examine the influence of expression levels of SDC-4, which is expressed in various types of cells, on clinical effect of WT1 peptide vaccination from multiple pathophysiological aspects. Further studies should be needed to address this issue.

Regarding the two risk factors in Table 1 and Supplementary Table 3, low-performance status is a common risk factor for poor prognosis of GBM, and the past chemotherapy might dampen immunological competence that supports WT1 peptide-based immunotherapy. On the other hand, although old age is a common risk factor for poor prognosis of GBM as well as low-performance status, no association between OS and age was detected. Since WT1 immunotherapy is a very mild therapy without significant adverse effects (only skin erythema at the vaccine injection sites) that give the organ damages, almost all patients, regardless of ages, are tolerable to the WT1 immunotherapy and may be effective to immunotherapy. On the other hand, since chemotherapy has strong adverse effects, the patients become nontolerable to it as the patients’ ages increase, resulting in the decrease in clinical outcome. Therefore, age is not important prognostic factor in immunotherapy, but PS, which reflects the patients’ immune conditions, is a very important prognostic factor. Repeated chemotherapy gives the damages to the immune system and thus decreases the clinical effects of the following immunotherapy. Therefore, the results that previous chemotherapy but not age is a bad prognostic factor are reasonable.

To our knowledge, only one previous study [56] involved experiments similar to ours; those authors reported biomarkers that predicted the outcome of vaccination with four kinds of HLA-A-restricted peptides in patients with conventional therapy-resistant prostate cancer. In particular, they identified *LRRN3*, *PCDH17*, *HIST1H4C* and *PGLYRP1*, but not *SDC-4*, as biomarkers that discriminated between long- (>900 days) and short-term (≤900 days) survivors. The discrepancy in the markers identified in our study and theirs, which used a different peptide vaccine, implies that different targeted TAAs lead to different immune responses in patients. This might mean that these different immune responses are dependent upon whether T cells, DCs and MDSCs or other cell types are major players in the immune reaction, and that the major players are determined by differences in the targeted TAAs and/or types of malignancies.

Since sample size of the present study was not so large, the processes to identify the *SDC-4* as a biomarker were divided into the two: discovery and validation processes. The candidate genes identified in the discovery process using 30 patients were validated in the next process using different 23 patients in a validation set, resulting in the identification of *SDC-4* alone as a biomarker to predict clinical outcome of WT1 peptide vaccine. We wish to perform a prospective study composed of a larger sample size in the near future, and then we may obtain a more definite finding that supports the conclusion of the present study. In addition, a large sample size-based study may give us an opportunity to find another promising biomarker for WT1 peptide vaccination, and a combination of *SDC-4* and the other biomarkers may become a more precise predictor for clinical outcome of the therapy.

## Conclusion

In this study, among patients with glioblastoma who received WT1 peptide vaccine, low *SDC-4* expression level in PBMCs prior to vaccination was a prognostic factor for long-term survivors. This result was compatible with previously reported immune-suppressive functions of SDC-4 expressed on T cells. These findings suggested that *SDC-4* might be useful not only as a biomarker to select patients who would respond to the immunotherapy but also as a target to improve the effect of the immunotherapy.

**Table 1.** **Patients’ characteristics in a discovery set.**

**Characteristic**	**Short OS (n = 15)**	**Long OS (n = 15)**	**Hazard ratio 95% CI**		**p-value^†^**
			**Lower**	**Upper**	
Age (years):					
– Mean (range)	58 (25–67)	46 (20–75)	0.07	2.76	0.36
Gender:					
– Male (%)	9 (60)	11 (73)	0.35	3.88	0.93
KPS:					
– Mean (range)	75 (45–95)	80 (55–100)	0.005	0.50	0.009*
Surgical treatment (%)	13 (87)	15 (100)	0.15	11.7	0.90
Chemotherapy, n (%)	14 (93)	11 (73)	1.26	26.5	0.021*
RT before vaccination (Gy):					
– Average (SD)	67 (22.3)	61 (8.7)	0.03	13.3	0.96

^†^Cox proportional hazard regression with the OS from vaccination started as the time variable was used.

*Statistical significance (p < 0.05).

KPS: Karnovski performance status; OS: Overall survival; RT: Radio therapy (total absorbed dose); SD: Standard deviation.

**Table 2.** **Thirty-two candidate genes that differentially expressed between long- and short-term survivors in peripheral blood mononuclear cells prior to WT1 peptide vaccination in 30 glioblastoma multiforme patients in a discovery set.**

**Gene**	**Fold change^†^**	**p-value^‡^**
*TLR10*	0.85	0.051
*KLHDC8B*	0.80	0.018
*RALGPS2*	0.80	0.017
*CD79B*	0.71	0.029
*TNFAIP8L2*	0.69	0.026
GCNT2	0.65	0.017
*EVA1*	0.64	0.022
*CETN3*	0.59	0.013
*ROGDI*	0.58	0.012
*CD82*	0.57	0.004
*IL17RA*	0.55	0.009
*TNFSF14*	-1.02	<0.001
*CST1*	-0.90	0.005
*FBXO32*	-0.90	0.021
*ULBP2*	-0.88	0.028
*OASL*	-0.88	0.004
*PHLDA1*	-0.88	0.002
*FASLG*	-0.86	0.028
*XAGE5*	-0.83	0.002
*SDC4*	-0.80	0.024
*ZNF659*	-0.79	0.003
*SMAD7*	-0.69	0.011
*SLC7A5*	-0.68	0.008
*VPS37B*	-0.66	<0.001
*HPGD*	-0.65	0.018
*MLF1*	-0.65	0.012
*SKI*	-0.63	0.006
*EXPH5*	-0.63	0.009
*ZC3H12A*	-0.61	0.007
*UAP1*	-0.57	0.015
*CCNT1*	-0.53	<0.001
*ITGA5*	-0.52	0.002

^†^Log_2_(long/short).

^‡^Two-tailed Welch's t-test.

**Table 3.** **Verification of 32 candidate genes by quantitative RT-PCR.**

**Gene**	**Transformation**	**Normal distribution**	**Statistical method**	**Fold change^†^**	**p-value**	**Result**
*EXPH5*	Square root	True	Left-sided Student t-test	-0.34	<0.001*	Verified
*ZC3H12a*	Failed	False	Left-sided Mann–Whitney U-test	-1.23	0.004*	Verified
*VPS37B*	Not needed	True	Left-sided Student t-test	-1.19	0.005*	Verified
*PHLDA1*	Square root	True	Left-sided Student t-test	-1.33	0.007*	Verified
*TNFSF14*	4th root	True	Left-sided Student t-test	-1.70	0.007*	Verified
*UAP1*	Square root	True	Left-sided Student t-test	-1.58	0.013*	Verified
*HPGD*	Cube root	True	Left-sided Student t-test	-0.68	0.016*	Verified
*SKI*	4th root	True	Left-sided Student t-test	-0.76	0.016*	Verified
*SDC4*	Logarithmic	True	Left-sided Student t-test	-0.35	0.023*	Verified
*FOXO32*	Logarithmic	True	Left-sided Student t-test	-2.02	0.023*	Verified
*SLC7A5*	Square root	True	Left-sided Student t-test	-0.97	0.026*	Verified
*ULBP2*	Logarithmic	True	Left-sided Student t-test	-2.04	0.030*	Verified
*CCNT1*	Cube root	True	Left-sided Student t-test	-0.60	0.031*	Verified
*FASLG*	Cube root	True	Left-sided Student t-test	-1.46	0.036*	Verified
*OASL*	Square root	True	Left-sided Student t-test	-1.04	0.039*	Verified
*SMAD7*	Square root	True	Left-sided Student t-test	-1.53	0.067	Excluded
*GCNT2*	Failed	False	Right-sided Mann–Whitney U-test	3.82	0.189	Excluded
*EVA1*	Not needed	True	Right-sided Student t-test	0.00	0.220	Excluded
*ITGA5*	Cube root	True	Left-sided Student t-test	-0.76	0.241	Excluded
*F3*	Failed	False	Left-sided Mann–Whitney U-test	-2.11	0.252	Excluded
*XAGE5*	Failed	False	Left-sided Mann–Whitney U-test	-1.77	0.261	Excluded
*MLF1*	Square root	True	Left-sided Student t-test	-0.46	0.448	Excluded
*TLR10*	Failed	False	Right-sided Mann–Whitney U-test	4.73	0.491	Excluded
*CETN3*	Cube root	True	Right-sided Student t-test	0.05	0.523	Excluded
*CD82*	Square root	True	Right-sided Student t-test	-0.08	0.577	Excluded
*TNFAIP8L2*	Cube root	True	Right-sided Student t-test	-0.40	0.646	Excluded
*CD79B*	Square root	True	Right-sided Student t-test	0.45	0.653	Excluded
*IL17RA*	Cube root	True	Right-sided Student t-test	0.47	0.738	Excluded
*RALGPS2*	Not needed	True	Right-sided Student t-test	-0.20	0.744	Excluded
*KLHDC8B*	Logarithmic	True	Right-sided Student t-test	0.37	0.821	Excluded
*ZNF659*	Failed	False	Left-sided Mann–Whitney U-test	1.42	0.934	Excluded
*ROGD1*	Square root	True	Right-sided Student t-test	0.07	0.982	Excluded

^†^Log_2_(mean of gene expression levels in long-term survivors/mean of gene expression levels in short-term survivors).

*Statistical significance (p < 0.05).

**Table 4.** **Patients’ characteristics in a validation set.**

**Characteristic**	**Short OS (n = 11)**	**Long OS (n = 12)**	**Hazard ratio 95% CI**		**p-value^†^**
			**Lower**	**Upper**	
Age (years):					
– Mean (range)	48.9 (28–63)	50.8 (30–71)	0.14	5.48	0.90
Gender:					
– Male (%)	7 (58)	7 (63)	0.21	1.88	0.40
KPS:					
– Mean (range)	78 (50–100)	84 (50–100)	0.08	2.21	0.29
Surgical treatment (%)	10 (91)	12 (100)	0.06	19.9	0.86
Chemotherapy, No. (%)	9 (82)	10 (83)	0.11	2.12	0.28
RT before vaccination (Gy):					
– Average (SD)	57 (9.0)	59 (2.9)	0.005	2.54	0.13

^†^Cox proportional hazard regression with the OS from vaccination started as the time variable was used.

KPS: Karnovski performance status; OS: Overall survival; RT: Radio therapy (total absorbed dose); SD: Standard deviation.

**Table 5.** **Validation of 15 candidate genes using different 23 glioblastoma multiforme patients in a validation set.**

**Gene**	**Fold change^†^**	**p-value^‡^**	**Result**
*SDC4*	-1.85	0.020*	Validated
*HPGD*	-0.84	0.076	Not reproducible
*PHLDA1*	-0.59	0.154	Not reproducible
*VPS37B*	-1.81	0.269	Not reproducible
*OASL*	-0.21	0.485	Not reproducible
*TNFSF14*	0.27	0.555	Not reproducible
*FASLG*	3.62	0.602	Not reproducible
*UAP1*	2.75	0.608	Not reproducible
*SLC7A5*	1.82	0.679	Not reproducible
*FOXO32*	2.21	0.760	Not reproducible
*SKI*	3.80	0.844	Not reproducible
*EXPH5*	2.43	0.869	Not reproducible
*ULBP2*	2.65	0.953	Not reproducible
*ZC3H12a*	2.71	0.964	Not reproducible
*CCNT1*	1.09	0.975	Not reproducible

^†^Log_2_(long/short).

^‡^The same statistical methods as those in Table 3 were used.

*Statistical significance (p < 0.05).

**Executive summary**

**Background**

WT1 overexpresses in leukemia and a variety of solid tumors and performs an oncogenic function, and WT1 protein is one of the most superior pan-tumor-associated antigens.
Glioblastoma multiforme (GBM) is a malignant brain tumor with very poor prognosis, and once recurrence occurs, therapeutic options are limited.
In 2008, we reported the promising results of a Phase II clinical study of WT1 vaccination for recurrent or conventional therapy-resistant GBM patients.
In order to improve the clinical usefulness of WT1 peptide vaccine, it is important not only to enhance clinical efficacy but also to select the responders to WT1 peptide vaccine.
Novel methods to predict responders prior to WT1 vaccination were awaited.

**Materials & methods**

Peripheral blood mononuclear cells from 30 GBM patients prior to WT1 vaccination in a discovery set were subjected to cDNA microarray analysis, candidate genes that differentially expressed between long- and short-term survivors were selected, followed by verification with quantitative RT-PCR.
The filtrated candidate genes were validated for the correlation between gene expression levels and clinical effects using different 23 GBM patients of a validation set.

**Results**

Thirty-two differentially expressed genes (DEGs) were extracted by cDNA microarray analysis using 30 GBM patients in a discovery set, and 15 DEGs were verified by quantitative RT-PCR.
The 15 DEGs were validated using 23 patients in a validation set, and only *SDC-4* was identified as a biomarker for prediction of overall survival (OS).
Cut-off value of mRNA expression levels of *SDC-4* that discriminated between OS of ≥256 days (responders) and OS of <56 days (nonresponders) was 0.001, and the *SDC-4* expression levels of ≤0.001 and >0.001 predicted responders and nonresponders, respectively, with 70.5% sensitivity and 76.0% specificity.
One-year OS rates were 64.0 and 18.5% in *SDC-4*-low and -high patients, respectively.

**Conclusion**

*SDC-4* is a biomarker to predict clinical outcome for GBM patients treated by WT1 peptide vaccine.
