# Supplementary material for: Syndecan-4 as a biomarker to predict clinical outcome for glioblastoma multiforme treated with WT1 peptide vaccine
Source: Future Sci OA. 2016 Oct 3;2(4):FSO96. doi: 10.4155/fsoa-2015-0008 (PMC5241910; doi:10.4155/fsoa-2015-0008)
Supplement: Supplementary file 1 [file fsoa-02-96-s1.docx]

**Supplementary Table 1. Events and censors of patients**

|  | **Discovery set** | | | | **Validation set** | |
| --- | --- | --- | --- | --- | --- | --- |
|  | **screening** | | **verification** | | **validation** | |
|  | **short-OS** | **long-OS** | **short-OS** | **long-OS** | **short-OS** | **long-OS** |
|  |  |  |  |  |  |  |
| **Number** | 15 | 15 | 15 | 15 | 11 | 12 |
|  |  |  |  |  |  |  |
| **Censored** | 0 | 5 | 0 | 5 | 0 | 2 |
|  |  |  |  |  |  |  |
| **died of tumor progression** | 15 | 10 | 15 | 10 | 11 | 10 |
|  |  |  |  |  |  |  |
| **alive** | 0 | 5 | 0 | 5 | 0 | 2 |
