# Supplementary material for: Syndecan-4 as a biomarker to predict clinical outcome for glioblastoma multiforme treated with WT1 peptide vaccine
Source: Future Sci OA. 2016 Oct 3;2(4):FSO96. doi: 10.4155/fsoa-2015-0008 (PMC5241910; doi:10.4155/fsoa-2015-0008)
Supplement: Supplementary file 2 [file fsoa-02-96-s2.docx]

**Supplementary Table 2. Primer sequences for quantitative RT-PCR**

| Gene | AccessionNo | Forward primer | Reverse primer |
| --- | --- | --- | --- |
| *TLR10* | NM_030956 | AGATTGCTTTTGCCACCAAC | TCTCACATCTCCTTTTGATAGCC |
| *KLHDC8B* | NM_173546 | GTAGAGGCCTTCCTGATGGA | CCCCCAGAGCATACACCATA |
| *RALGPS2* | NM_152663 | TGGCTATCGAAGTCACATGAA | TTTTCTCCTGAGAACACCTTGAA |
| *CD79B* | NM_021602 | TGCTGCTGCTGCTCTCAG | CAAGGTGCTGAATCCTTTGG |
| *TNFAIP8L2* | NM_024575 | CTTGGACAGATGCCAGAGG | GAGAATCTCCCCTGCCCTA |
| *GCNT2* | NM_145655 | TGATGAGCATTTCTGGGTGA | CCAGGATGCATTTGGCATA |
| *EVA1* | NM_005797 | GGCCGAAAGAGCTCATAAAG | CAGAGACCTTTTTCTCTTGGTTG |
| *CETN3* | NM_004365 | ATTAAAGGTGGCAATGAGAGCCT | GCAACACGTCGCAAATTCCTCA |
| *ROGDI* | NM_024589 | GGGAGGACAAGCAGTGGA | GCTTGGCTCACATGGTTTCT |
| *CD82* | NM_002231 | GAAAGCAGAACCCGCAGA | CCAGTGCAGCTGGTCACA |
| *IL17RA* | NM_014339 | CATCCTGCTCATCGTCTGC | GCCATCGGTGTATTTGGTGT |
| *ITGA5* | NM_002205 | CCCATTGAATTTGACAGCAA | TGCAAGGACTTGTACTCCACA |
| *CCNT1* | NM_001240 | CTTACTTCATGGCAACCAACAG | CAAGCCAGGTGAATGCAGA |
| *UAP1* | NM_003115 | CAGGGGGCCATTTCATAGA | TCATTGGCATCCTTCAAGC |
| *ZC3H12A* | NM_025079 | ATCGATGGGAGCAACGTG | CCGCTCCAGAAACCAGTTC |
| *EXPH5* | NM_015065 | CGGCGTTTGATTTCAGTTTC | CTCTTTGTCTTCTGAAGTTTGCTG |
| *SKI* | NM_003036 | GAAGCAGGAGGAGAAGCTCAG | CCACGCGTAGGAACTCCA |
| *MLF1* | NM_022443 | TGAGAAGTGTTGGCCATGAG | GGCTGGACTTTGTTGAGGTT |
| *HPGD* | NM_000860 | CAGAAGACTCTGTTCATCCAGTG | TGTCCAGTCTTCCAAAGTGGT |
| *VPS37B* | NM_024667 | CCAGCTGACGGAGATGGT | TTGCTGGCAAGTGTCATTTC |
| *SLC7A5* | NM_003486 | GTGGAAAAACAAGCCCAAGT | GCATGAGCTTCTGACACAGG |
| *SMAD7* | NM_005904 | AAACAGGGGGAACGAATTATC | ACCACGCACCAGTGTGAC |
| *ZNF659* | NM_024697 | TGCAAGGTTGCTGTCAACTC | GGCTTCTAACATGGTTTTGTGC |
| *SDC4* | NM_002999 | GGCAGGAATCTGATGACTTTG | GCCGATCATGGAGTCTTCC |
| *XAGE5* | NM_130775 | GGGTGCAGCTGAGATTCAAG | TCCCCTGGACATCAGGACTA |
| *FASLG* | NM_000639 | TGGGGATGTTTCAGCTCTTC | TGTGCATCTGGCTGGTAGAC |
| *PHLDA1* | NM_007350 | CCTCCAACTCTGCCTGAAAG | TCGTCCCACTTCCTCAAGTC |
| *OASL* | NM_198213 | TTGCTATGACAACAGGGAGAAC | CACTGTCAAGTGGATGTCTCG |
| *ULBP2* | NM_025217 | CCGCTACCAAGATCCTTCTG | GGATGACGGTGATGTCATAGC |
| *FBXO32* | NM_148177 | GCAGCAGCTGAACAACATTC | CACAAAGGCAGGTCAGTGAA |
| *CST1* | NM_001898 | GAACAGCCAGAACTGCAGAA | GGATTTCACCAGGGACCTTC |
| *TNFSF14* | NM_003807 | AGCGAAGGTCTCACGAGGT | CGGTCAAGCTGGAGTTGG |
| *ACTB(Ctrl)* | X00351 | CCAACCGCGAGAAGATGA | CCAGAGGCGTACAGGGATAG |
