# Supplementary material for: Syndecan-4 as a biomarker to predict clinical outcome for glioblastoma multiforme treated with WT1 peptide vaccine
Source: Future Sci OA. 2016 Oct 3;2(4):FSO96. doi: 10.4155/fsoa-2015-0008 (PMC5241910; doi:10.4155/fsoa-2015-0008)
Supplement: Supplementary file 3 [file fsoa-02-96-s3.docx]

**Supplementary Table 3. *SDC-4* expression levels as an independent predictor**

| **variables** | **hazard ratio** | **95% CI** | | **p-value†** | |
| --- | --- | --- | --- | --- | --- |
|  |  | **lower** | **upper** |  |  |
| ***SDC-4* expression level** | 13.8 | 1.35 | 84.2 | 0.027 | * |
|  |  |  |  |  |  |
| **KPS** | 0.33 | 0.11 | 0.99 | 0.029 | * |
|  |  |  |  |  |  |
| **History of chemotherapy** | 2.48 | 1.05 | 6.77 | 0.049 | * |
|  |  |  |  |  |  |
| **Gender (Male)** | 0.56 | 0.28 | 1.13 | 0.09 |  |
|  |  |  |  |  |  |
| **Surgical Treatment** | 0.31 | 0.09 | 1.49 | 0.14 |  |
|  |  |  |  |  |  |
| **RT, Gy** | 0.11 | 0.001 | 5.15 | 0.27 |  |
|  |  |  |  |  |  |
| **Age** | 0.58 | 0.16 | 2.15 | 0.58 |  |

Abbreviations: CI, confidence interval; KPS, Karnovski performance status; RT, radio therapy (total absorbed dose)

†Cox proportional hazard regression models with the OS from vaccination started as the time variable were used.
